# Effect of a Wide Stance on Block Start Performance in Sprint Running

**DOI:** 10.1371/journal.pone.0142230

**Published:** 2015-11-06

**Authors:** Mitsuo Otsuka, Toshiyuki Kurihara, Tadao Isaka

**Affiliations:** Faculty of Sport and Health Science, Ritsumeikan University, 1-1-1 Nojihigashi, Kusatsu, Shiga, 525–8577, Japan; Semmelweis University, HUNGARY

## Abstract

This study aimed to clarify the effect of widened stance width at the set position during the block start phase in sprint running on kinematics and kinetics at the hip joint and block-induced power. Fourteen male sprinters volunteered to participate in this study. They performed three block-start trials with a normal stance width (25 ± 1 cm, normal condition) and a widened stance width (45 ± 2 cm, widened condition) at the set position. The block start movements were recorded at 250 Hz with high-speed cameras and the ground reaction forces at 1250 Hz with force plates. During the block phase in the widened condition, the hip abduction and external rotation angles in both legs were significantly larger and smaller, respectively, than those in the normal condition. The positive peak value of the hip power in the rear leg was significantly greater in the widened condition than that in the normal condition. However, no significant difference was seen in the normalized block-induced power between the widened and normal conditions. We conclude that a widened stance width at the set position affects the hip-joint kinematics and rear hip power generation during the block start phase, but no effect on the block-induced power when considering sprinting performance during the whole block start phase.

## Introduction

During the pushing phase on starting blocks, the average value of external power in an anterior direction to translate the whole-body centre of mass (COM; hereafter, block-induced power) is important for a great performance in the 100-m dash [1,2]. This is associated with the extension of front and rear legs. Sprinters are permitted to adjust the anteroposterior position and inclination of both starting blocks in accordance with the regulations for athletes [3]. Several previous studies have clarified the effect of different body postures at the set position on the subsequent sprinting motion during the block start phase [4–8]. For instance, relative to the elongated start, bunched and medium starts shorten the pushing duration during the pushing phase on the starting block, thereby shortening the subsequent sprinting time at 5 m and 10 m [7,8]. This may be due to the optimal position of body segments, which enhances the power generation of the lower limbs and block-induced power during the block start phase [4,5].

Sprinters extend both legs during the block start phase from a bending position [2,9]. Peak extension angular velocity of the front and rear hips, contributes to the block-induced power during the block start phase, unlike that of the knee and ankle [2]. This rapid hip extension might be associated with greater power generation at the hip joint, thereby providing greater block-induced power during the block start phase. The hip power generation is larger than those at the knee and ankle of the front and rear legs during the block start phase [5]. These findings suggest that the hip extensor kinetics is a key contributor to high block-induced power. This leg extension motion during the block start phase is similar to the double-legged squat motion [10]. Biomechanical analysis of the squatting motion has revealed that lengthening the mediolateral distance between feet (140% of shoulder width) contributes to stronger isometric contractions in lower limb muscles [11]. This widened stance width enhances the mean electromyography value of the gluteus maximus during a squat [12–14]. The stance width at the set position of the block start has been reported to be 23 ± 1 cm [15]; this is shorter than the stance width in previous studies on widened stance width in squatting [11–14]. These studies may indicate that a widened stance width during the block start phase would enhance block-induced power during the block start phase attained by a greater hip joint power. Nevertheless, sprinters may not be able to select enough stance width at the set position for greater block-induced power using the current competition blocks [3]. For instance, the mediolateral width of starting blocks in overall dimension is 30 cm (RM-150, Seiko, Tokyo, Japan). This width is narrower than the stance width in squats used to enhance muscle strength [11].

Thus, further investigation of squatting to block start is required to elucidate the effect of a wide stance width on block-induced power. This information would help in reconsidering block start rules [3], developing new starting block designs, and aiding sprinters in the appropriate placement of starting blocks in the future. This study aimed to clarify the effect of widened stance width at the set position during the block start phase on hip kinematics, hip kinetics and block-induced power. Our hypotheses were as follows: 1) widened step width during the block start phase would lead to sprinters changing their hip position and enhance block-induced power attained by the high hip power generation, and 2) widened step width during the block start phase would affect the relationship between changes in block-induced power and those in the hip power generation.

## Materials and Methods

### Participants

Fourteen male sprinters (mean ± standard deviation [SD]; age: 21.1 ± 1.2 years, body mass: 64.5 ± 3.9 kg, height: 1.76 ± 0.04 m) volunteered to participate in this study. Three participants were international-level sprinters, who were finalists in the National Championship. All participants were sprint specialists with training experience of ≥6 years (8.4 ± 2.4 years), and the average personal best 100-m time was 10.99 ± 0.40 s (range: 10.21–11.65 s).

The experimental protocol was approved by the Research Ethics Committee Involving Living Human Subjects at Ritsumeikan University (BKC-human-2011-011). Each participant provided written informed consent before study participation. The individual in this manuscript has given written informed consent (as outlined in PLOS consent form) to publish these case details.

### Experimental procedure

**Fig 1:**
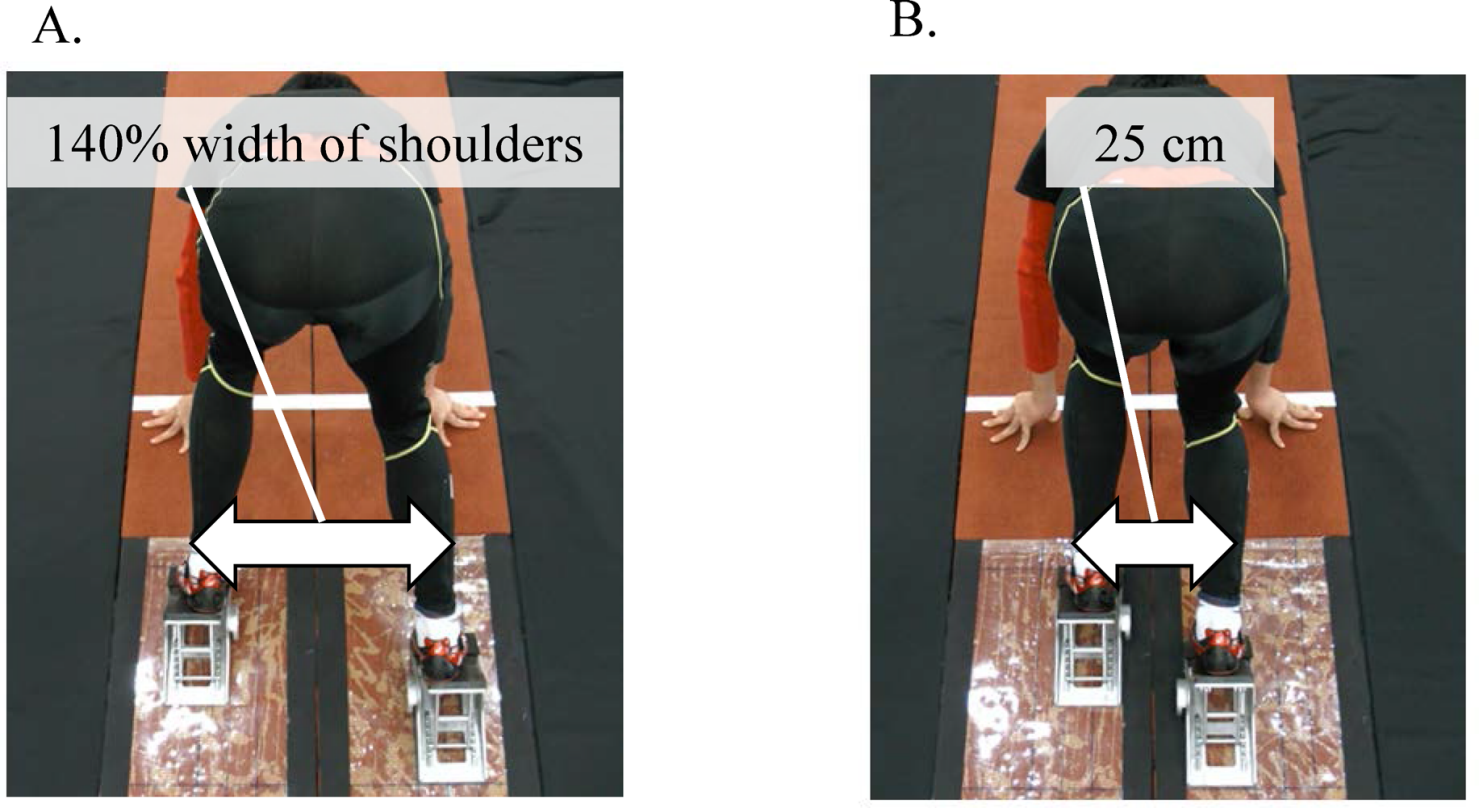
A postero-superior view of the set position during the block start phase. (A) Set position in the widened condition. (B) Set position in normal condition.

The participants were asked to perform a 10-m sprint on an indoor track, exerting maximum effort from a crouching position, with a widened (45 ± 2 cm, widened condition, Fig 1A, S1 Video) and normal (25 ± 1 cm, normal condition, Fig 1B, S2 Video) stance width (mediolateral distance between midpoints of first and fifth metatarsals of feet). The participants were instructed to start after a gun signal by a starter [3]. The stance width in the widened condition corresponded to 140% of shoulder width during a squat at widened stance width [11,13]. Each participant performed three trials each with normal and widened stance width using their own spike shoes, and the order of trials in both conditions was randomized. To reduce the effect of body size and crouching position type on block performance, the anteroposterior distance of the starting blocks was adjusted to 12% of each participant’s height (21 ± 1 cm) so as to be corresponded with the bunched start [7,8,16]. The anteroposterior distance between the feet in a bunched start is closer to that of a squat motion relative to those of medium and elongated starts. Throughout the experiment, the block angles of front and rear legs were set at 40° and 42°, respectively [4]. The starting blocks were securely anchored to the synthetic track surface on two separate force plates (0.40 m × 0.60 m; TF-4060-B; Tech-Gihan, Inc., Kyoto, Japan). Before the experimental trials, an appropriate 15-min warm-up including jogging and stretching and at least three sprints in each stance width condition were performed.

### Data collection

Data on the participants’ sprinting movement and ground reaction force (GRF) during the block start phase were captured simultaneously. A total of 36 retro-reflective markers sized 12 mm were attached on the pelvis and lower limbs, and the three-dimensional locations of the markers were recorded using a 16-camera motion capture system (Raptor-E digital; Motion Analysis Corporation, Santa Rosa, CA, USA) sampling at 250 Hz. The GRF data from legs were separately measured by positioning a total of 2 force plates (TF-4060-B; Tech-Gihan, Inc., Kyoto, Japan), arranged in two rows of two each, sampling at 1250 Hz. The sprinting time was measured based on signals from the gun (EP; Molten Inc, Hiroshima, Japan) and photocell (E3G-R13; Omuron Inc., Kyoto, Japan) set at 2.0-m mark, and were synchronized with the GRF data.

### Data processing

**Fig 2:**
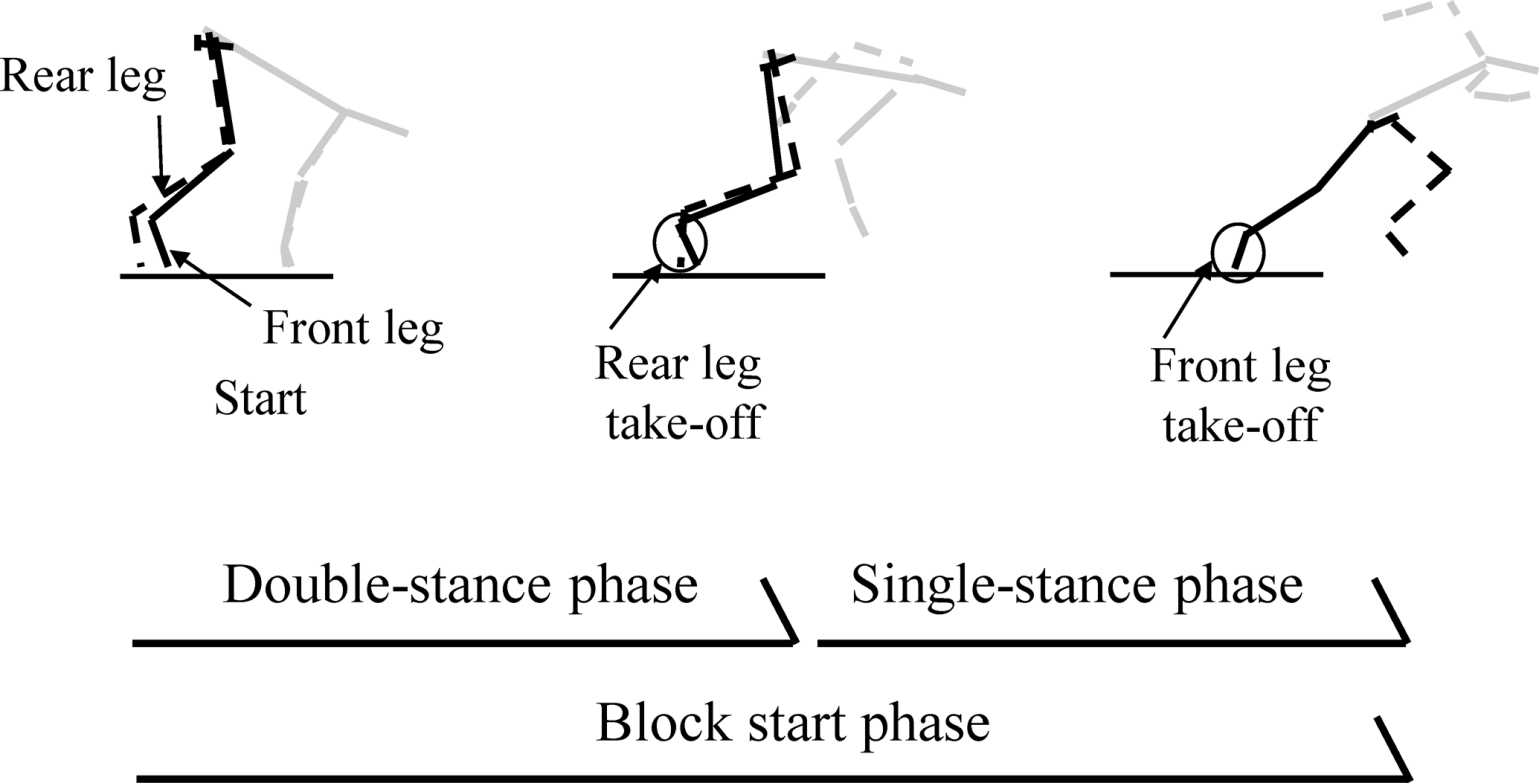
Definition of the double-leg and single-leg stance phases during the block start phase. The block start phase was divided into double-leg and single-leg stance phases based on the instant of rear-leg take-off. The black solid line represents the pelvis and front leg, the black dashed line represents the rear leg, and the grey line represents the other segments that were not analyzed in this study.

GRF data were not filtered [17]. We used a vertical GRF threshold of 10 N to determine the instant of take-off from the starting blocks. We then used these readings to divide the block start phase into the double-stance and single-stance phases (Fig 2).

The marker trajectory data were filtered using a fourth-order, zero-lag, low-pass Butterworth filter, and the cut-off frequency was set at 10 Hz [15]. A 7-segment rigid body model including the pelvis, thighs, shanks, and feet was created. The segmental data were calculated using mass properties based on cadavers [18]. The locations of the center of mass and inertial properties were obtained using a mathematical model [19].

For comparison between different sprinters, the dimensionless [20] normalized block-induced power was considered as the best indicator of sprinting performance during the block start phase (hereafter, normalized block-induced power) [1,2]. So as to clarify the relationship between changes in block-induced power and those in the hip power generation, the dimensionless normalized block-induced power (*P*_*N*_) was calculated as follows:

$$P_{N}\;=\;\bar{P}/(m\;g^{3/2}l^{1/2})$$ (1)

where *m* is the mass of the sprinter, *g* is the acceleration due to gravity, and *l* is the leg length of the sprinter. Block-induced power ($\bar{P}$) was calculated as follows [1,2]:

$$\bar{P}\;=\;m\left(v_{f}^{2}-v_{i}^{2}\right)/(2\;\Delta t)$$ (2)

where *v*_*i*_ and *v*_*f*_ are the anteroposterior velocities of COM at the start (here the *v*_*i*_ = 0 m/s) and end of the block start phase, respectively, and *Δt* is the pushing duration during the block start phase. Here, the *v*_*f*_ is equal to the GRF impulse of the anteroposterior component normalized by body mass during the block start (hereafter, normalized anteroposterior impulse, *I*_*N*_). The *I*_*N*_ was calculated as follows [21]:

$$I_{N}\;=\;\int_{t_{1}}^{t_{2}}\;{apGRF}_{Sum}\;dt/m$$ (3)

where *t*_*1*_ and *t*_*2*_ are the times at which force application begins and ends, respectively, and *apGRF*_*Sum*_ is the anteroposterior component of the sum of GRFs from the legs during the block start phase. The normalized front-block-induced power, normalized rear-block-induced power, and normalized anteroposterior impulses in front and rear legs were calculated, after adjusting for Eqs (1), (2) and (3) based on the number of force plates. Mean value of anteroposterior and mediolateral accelerations of COM during the block start phase (mean anteroposterior acceleration ($\bar{apCOMacc}$) and mean mediolateral acceleration ($\bar{mlCOMacc}$), respectively) was calculated as follows:

$$\bar{apCOMacc}=\bar{{apGRF}_{Sum}}/m$$ (4)

$$\bar{mlCOMacc}=\bar{{mlGRF}_{Sum}}/m$$ (5)

where $\bar{{apGRF}_{Sum}}$ and $\bar{{mlGRF}_{Sum}}$ are anteroposterior and mediolateral components of sum of GRFs vector from the legs, respectively. Reaction time, pushing durations in front and rear legs during the block start phase, sprint times up to 2.0 m with the reaction time were calculated.

Hip angle, moment and power data were calculated using an algorithm in Visual 3D (v4.86.0; C-motion, Inc., Germantown, MD, USA). The locations of the center of rotation of the hip [22], knee [23], and ankle [24] were estimated from anatomical landmarks using a predictive approach. In each pelvic [24], thigh [24], shank [23], and foot [24] anatomical coordinate system, the *x*-axis represented the extension–flexion axis of segment rotation, the *z*-axis of the distal frame represented the external–internal rotation axis, and the axis orthogonal to the previous two at any given instant in time (*y*-axis) represented the abduction–adduction axis. Hip extension, abduction, and external rotation angles of front and rear legs were calculated using a hip joint coordinate system based on the *x*-*y*-*z* rotation sequence [24]. Knee and ankle joint coordinate systems were created using methods of Grood and Suntay [23] and Wu et al. [24], respectively. The hip extension moments of the front and rear/swing legs were calculated using a standard inverse dynamics approach [25], and were normalized by body mass (*M*_*hip*_): positive value indicates extension moment whereas negative value indicates flexion moment. The hip extension angular velocity (*ω*_*hip*_) was calculated by Winter’s method: positive value indicates extension angular velocity whereas negative value indicates flexion angular velocity [25]. The hip joint power (*P*_*hip*_) for all sprinters was calculated as follows:

$$P_{hip}\;=\;M_{hip}\omega_{hip}$$ (6)

Positive power results when the hip joint moment acts in the same direction as the angular velocity of the hip joint (concentric action). Negative power results when the hip joint moment acts in the opposite direction as the angular velocity of the hip joint (eccentric action). We used cubic spline interpolation to normalize these values with respect to time, with 100% representing the time of the block start phase.

Changes in normalized block-induced power were calculated (ΔNormalized block-induced power), and changes in the normalized block-induced power by legs as well as changes in the mean positive value of hip joint power in the widened condition relative to normal condition were calculated at each double- and single-stance phases (ΔNormalized block-induced power, ΔNormalized front-block-induced power, ΔNormalized rear-block-induced power, ΔFront-hip power generation, and ΔRear-hip power generation, respectively). These changes of variables in the widened condition relative to those normal condition (*Δvar*) were calculated as follows:

$$\Delta var\;\left[\%\right]\;=\;({var}_{W}/{var}_{N}-1)\;*\;100$$ (7)

where *var*_*W*_ and *var*_*N*_ are mean values of time-series variables during each phase in the widened and normal conditions, respectively. Normalized block-induced powers in the two conditions to calculate ΔNormalized block-induced, ΔNormalized front-block-induced and ΔNormalized rear-block-induced powers were calculated adjusting Eqs (1), (2) and (3) based on the phase and number of force plates. For instance, when calculating the block-induced power for ΔNormalized front-block-induced power during the double-stance phase in the widened condition, *v*_*i*_ is 0 m/s, *v*_*f*_ is the anteroposterior impulse on a force plate exerted by the front foot divided by body mass during the double-stance phase, and *Δt* is the duration during the double-stance phase in the widened condition.

### Statistical analysis

For all parameters, the mean value of all three trials in each condition was used for further analysis [2]. All parameters are shown as mean ± SD. We calculated the intraclass correlation coefficient (ICC(1,3)) for all parameters among three trials. The Lilliefors test was used to assess normality of variables. In the case of normally distributed samples, paired t-tests were used to assess the differences in the variables, and in the remaining, the Wilcoxon test was used for paired samples. Pearson’s correlation coefficient (r) was used to assess the relationships of changes in variables. Two-way repeated-measure analysis of variance and the post-hoc tests were used to assess the different effects of two conditions and legs on the variables (condition [widened and normal] x leg [front and rear]). The level of significance was set at P < 0.05.

## Results

**Fig 3:**
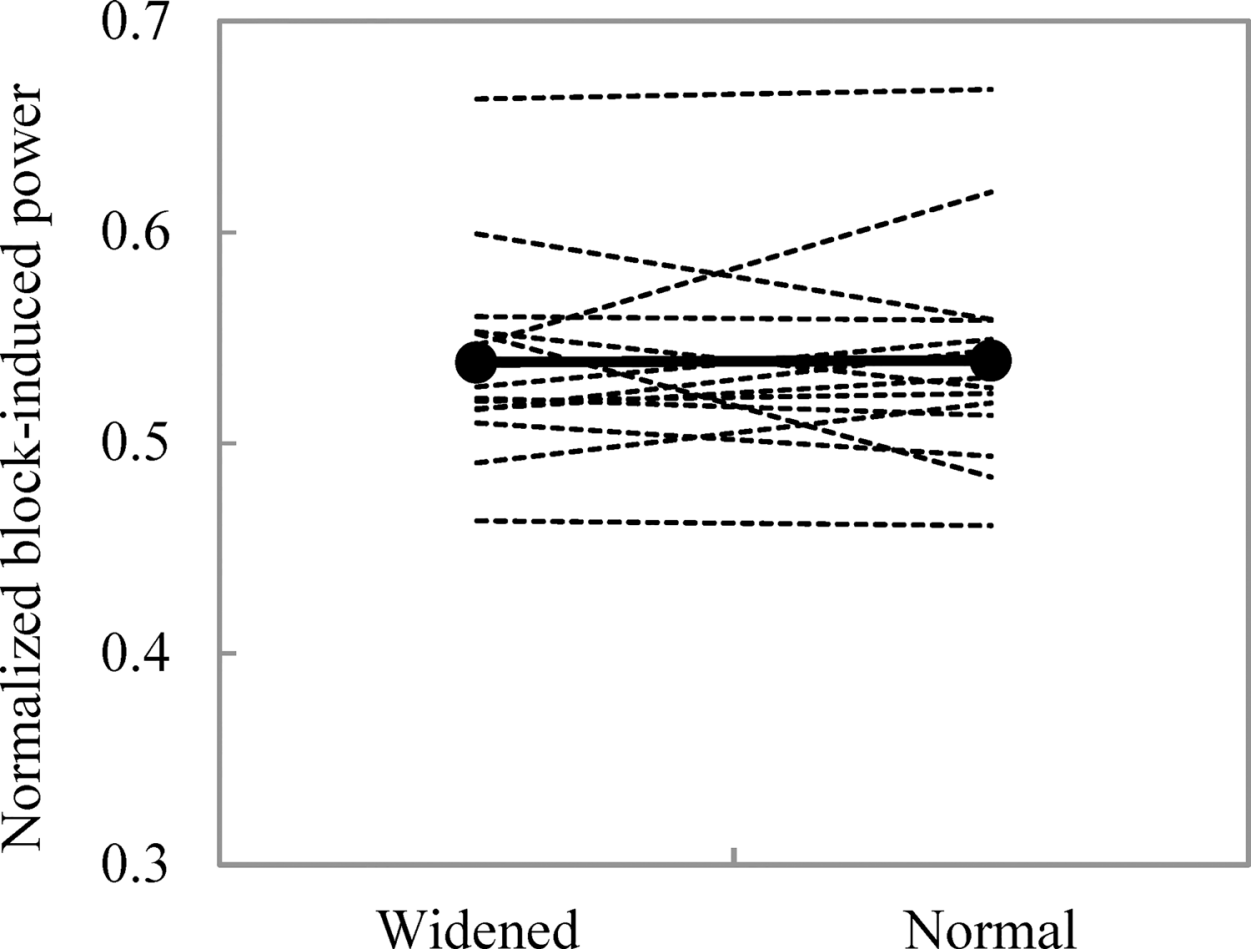
Normalized block-induced power in widened and normal conditions. The bold solid line indicates the mean value of normalized block-induced power in all participants, and the thin dashed lines indicate the normalized block-induced power in each participant (n = 14).

Table 1 shows the results of sprinting performance. Of the study participants, no significant difference in normalized block-induced power was seen between the widened and normal conditions (Fig 3). No significant difference was seen in the anteroposterior impulse, mean anteroposterior acceleration, reaction time, and duration of the block start phase between the two conditions. No significant difference was seen in the sprint time up to 2.0 m between the two conditions. All ICC(1,3) in sprinting performance exceeded 0.700 expect for reaction time (widened condition: ICC(1,3) = 0.626; normal condition (ICC(1,3) = 0.355).

**Table 1:** Mean ± SD, range and reliability of sprinting performance during the block start phase and the subsequent sprinting time in widened and normal conditions.

Parameter	Generator	Widened condition			Normal condition			Δ%	
		Mean ± SD	Range	ICC	Mean ± SD	Range	ICC	Mean ± SD	Range
Normalized block-induced power	Both legs	0.543 ± 0.051	0.461–0.663	0.886	0.539 ± 0.053	0.461–0.668	0.891	0.9 ± 5.3	−8.5–10.8
	Front leg	0.219 ± 0.023†	0.187–0.259	0.826	0.241 ± 0.042†	0.183–0.344	0.864	−7.5 ± 13.7†	−24.8–27.9
	Rear leg	0.130 ± 0.030*	0.091–0.191	0.954	0.113 ± 0.034	0.042–0.161	0.875	22.6 ± 37.0	−9.7–126.7
Anteroposterior impulse (Ns/kg)	Both legs	3.20 ± 0.20	2.94−3.57	0.961	3.20 ± 0.18	2.98–3.53	0.938	−0.1 ± 2.3	−5.8–3.4
	Front leg	2.03 ± 0.11* †	1.89–2.25	0.924	2.14 ± 0.14†	1.95–2.41	0.929	−4.6 ± 5.9†	−14.1–5.5
	Rear leg	1.15 ± 0.16*	0.89–1.40	0.910	1.05 ± 0.22	0.55–1.30	0.705	12.1 ± 18.7	−13.1–60.2
Pushing duration during the block start phase (s)	Both legs	0.330 ± 0.025	0.292–0.368	0.952	0.334 ± 0.031	0.298–0.420	0.946	−0.9 ± 4.5	−12.4–4.1
	Front leg	0.330 ± 0.025†	0.292–0.368	0.952	0.334 ± 0.031†	0.298–0.420	0.946	−0.9 ± 4.5	−12.4–4.1
	Rear leg	0.180 ± 0.023	0.144–0.224	0.896	0.175 ± 0.034	0.132–0.276	0.932	3.7 ± 8.7	−18.8–13.6
Mean anteroposterior acceleration (m/s^2^)	―	9.73 ± 0.59	8.73–10.82	0.843	9.65 ± 0.72	8.10–11.03	0.923	1.0 ± 4.6	−6.2–12.8
Mean mediolateral acceleration (m/s^2^)	―	−0.70 ± 0.47*	−1.56–−0.14	0.927	−0.57 ± 0.41	−1.52–−0.05	0.924	50.0 ± 65.1	−43.5–202.0
Reaction time (s)	―	0.180 ± 0.023	0.144–0.224	0.626	0.179 ± 0.016	0.154–0.206	0.355	0.6 ± 9.0	−16.7–18.6
Sprint time up to 2 m (s)	―	0.810 ± 0.043	0.738–0.906	0.836	0.797 ± 0.046	0.734–0.900	0.883	1.7 ± 5.1	−4.8–15.7

*Significant difference from normal condition (P < 0.05).

†,§ Significant difference from rear leg (P < 0.05).

**Fig 4:**
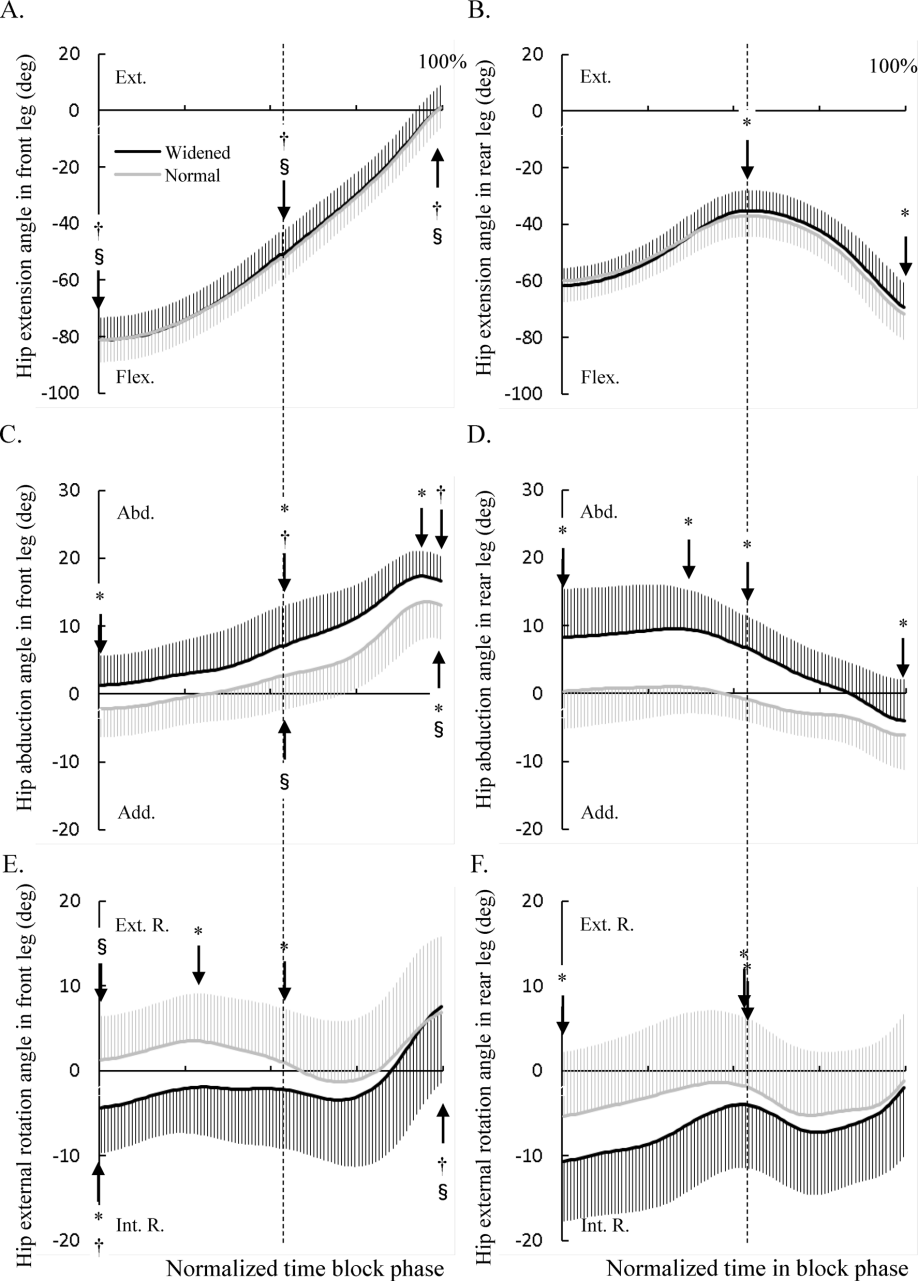
Changes in the hip angles during the block start phase normalized with respect to time. (A) Hip extension angles in front leg. (B) Hip extension angle in rear leg. (C) Hip abduction angle in front leg. (D) Hip abduction angle in rear leg. (E) Hip external rotation angle in front leg. (F) Hip external rotation angle in rear leg. The black and grey lines indicate the mean (bold) ± SD (thin) of the time-series data of the front and rear/swing legs in widened and normal conditions. These angles at the initial and end instants of double- and single-leg phases and at the instant when the peak value occurs were compared between the two conditions. Significant differences between the two conditions are shown as * (P < 0.05). Significant difference between the front and rear legs in widened condition are shown as † (P < 0.05) and that in normal condition are shown as § (P < 0.05). The vertical dashed line indicates the instant of rear leg take-off during the block start phase.

The hip extension angle in the front leg was not significantly different between widened and normal conditions during the double- and single-stance phases (Fig 4A). In contrast, the hip extension angle of the rear leg in the widened condition was significantly larger than that in the normal condition at the end of double-stance phase and the subsequent maximum extension angle (Fig 4B). The hip abduction angle in both legs in the widened condition was significantly larger than that in the normal condition during the block phase (Fig 4C and 4D). The hip external rotation angle of both legs in the widened condition was significantly lesser than that in the normal condition during the double-stance phase (Fig 4E and 4F).

**Fig 5:**
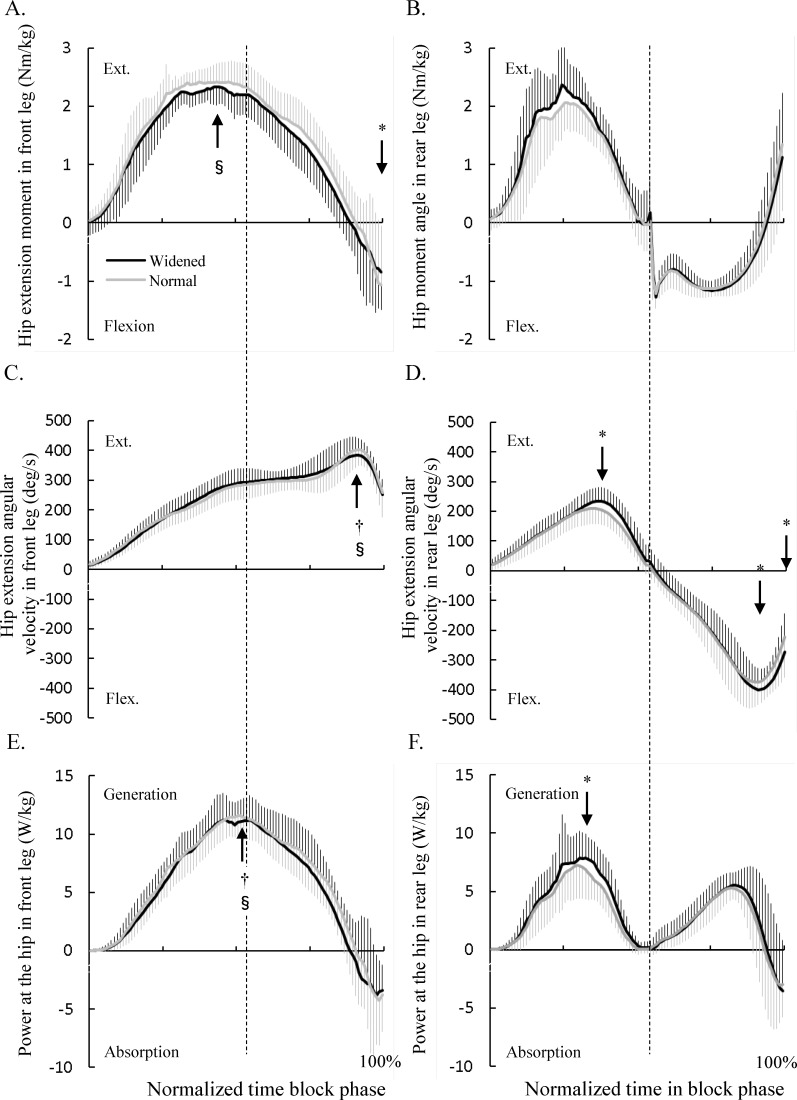
Changes in the hip moment, angular velocity, and joint power normalized with respect to time. (A) Hip extension moment in front leg. (B) Hip extension moment in rear leg. (C) Hip extension angular velocity in front leg. (D) Hip extension angular velocity in rear leg. (E) Hip joint power in front leg. (F) Hip joint power in rear leg. The black and grey lines indicate the mean (bold) ± SD (thin) of the time-series data of the front and rear/swing legs in widened and normal conditions. These angles at the initial and end instants of double- and single-leg phases and at the instant when the peak value occurs were compared between the two conditions. Significant differences between the two conditions are shown as * (P < 0.05). Significant difference between the front and rear legs in widened condition are shown as † (P < 0.05) and that in normal condition are shown as § (P < 0.05). The vertical dashed line indicates the instant of rear leg take-off during the block start phase.

No significant differences were seen in the hip extension moments in both legs during the double-stance phase (Fig 5A and 5B), while the hip extension moment in the widened condition was less than that in normal condition at the end of the single-stance phase. While no significant differences were seen in the hip extension angular velocity in the front leg during the block start phase (Fig 5C), the peak values of the hip extension and flexion angular velocity was significantly greater in the widened condition than that in the normal condition (Fig 5D). While no significant differences were seen in the hip power of the front leg between two conditions (Fig 5E), the positive peak value of the hip power of the rear leg in widened condition was significantly larger than that in the normal condition during double–stance phase (Fig 5F).

ΔNormalized block-induced power during the block start phase significantly related to that during the double-stance phase (r = 0.668; P < 0.01), but did not significantly relate to that during the single-stance phase (r = −0.021; n.s.). During the double-stance phase, this ΔNormalized block-induced power significantly related to the ΔNormalized rear-block-induced power (r = 0.793; P < 0.01), but did not significantly relate to the ΔNormalized front-block-induced power (r = 0.390; n.s.). During the double-stance phase, ΔNormalized rear-block-induced power significantly related to the ΔRear-hip power generation (r = 0.947; P < 0.01).

## Discussion

This study aimed to clarify the effect of widened stance width at the set position during the block start phase in sprint running on kinematics and kinetics at the hip joint, and block-induced power. During the block phase, the hip abduction and external rotation angles in both legs and peak hip power generation in rear leg in the widened condition were significantly changed compared to those in the normal condition. However, in the widened condition, no significant changes in normalized block-induced power were seen compared to that in the normal condition. This suggests that the response to widened stance width on the normalized block-induced power was not remarkable.

Previous studies have reported a significant effect of body posture in the sagittal plane on block performance [5,7,8]. A lower block angle (40°) leads sprinters to a 3.6% higher block-induced impulse than a higher block angle (65°) [5]. Other previous study has reported that the duration during the block start phase of the elongated start was 9.8% and 11.6% longer than that of medium and bunched starts, respectively [7]. In this study, the hip abduction and internal rotation angles of the front and rear legs during the double-stance phase were larger with a widened stance width than with a normal stance width; therefore, there was a change in the body position. However, no significant difference was seen in the sprinting performance, including the normalized block-induced power during the block start (−0.9 ± 5.2%), between the widened and normal conditions. These suggest that the changes in the set position that are related to the stance width have a lesser effect on the block performance relative to those related to the anteroposterior position and block angles.

We focused specifically on the hip power generation, which is considered as the key power for enhancing normalized block-induced power in lower limbs, rather than the power at the knee and ankle [2,5]. During the double-stance phase, the hip joint in the front and rear legs were generated power to induce hip extension, and during the single-stance phase, the hip in the rear leg was generated power to induce hip flexion. This was corresponding with the findings of the previous studies [5,9]. When the stance width is increased from narrow to wide during the squatting motion, the hip extensor muscle activity [12–14,26] and the maximum strength in lower limbs [11] increase. Demura et al. [11] compared the leg muscle strength between narrow (5 cm) and wide (140% width of the shoulders) stance widths in a squat. They found that the exerted maximum lower limb force in wide stance width was greater than that in narrow stance width. Similarly, in this study, the peak power generation at the hip in the rear leg was greater during the double-stance phase in the widened condition than that in the normal condition. This was due to enhancing the hip extension angular velocity in the rear leg. During the double-stance phase in the widened condition, hip in the rear leg was abducted and internally rotated, which was not the case in the normal condition. Perhaps this changed a property of the muscle contraction in hip extensor muscles during the double-stance phase and enhanced the muscle’s contraction velocity, which associates with the hip extension angular velocity, in the widened condition. In addition, the ΔRear-hip power generation was significantly associated with the ΔRear-block-induced normalized block-induced power, indicating that our second hypothesis was accepted in the rear leg. This suggests that those who preferred a widened stance width at the set position could increase the hip power generation in the rear leg by the optimal hip position and could enhance the normalized block-induced power during the double-stance phase. These findings supported the previous study demonstrating the importance of the rear leg [2].

However, when considering sprinting performance during the whole block start phase, no significant difference was seen in all sprinting performances between the two conditions. The block start phase involves the single-stance phase in which sprinters have to generate power with single-leg stance, in contrast to the squat motion. Therefore, it can be considered that during the single-stance phase in the widened condition, sprinters must push the block to a different direction relative to the normal condition. Indeed, mean mediolateral acceleration was less in the widened condition than in the normal condition, suggesting that the sprinter’s COM leaned toward the first step (rear leg) side during the single-stance phase. Thus, our first hypothesis in this study was rejected and did not correspond with the findings for the squatting position [11].

There are two limitations in this study. First, the number of combinations among stance width, block angles, and anteroposterior distance were limited for the starting blocks. The standardized set up was prepared with block angles and anteroposterior distance between the blocks. Therefore, each sprinter probably was not allowed to create their individual optimal set up for the starting blocks and probably could not perform their best block start in the normal condition. Moreover, the stance width in block start was not normalized by each participant’s body characteristics in the normal or widened conditions. We did not perform normalizations by body characteristics because the same stance width is conventionally used for all sprinters in competitive races, which is corresponded with that in normal condition. These might affect sprinting performance during the block start phase, changes in variables in the widened condition relative to the normal condition, and reliability of the results. However, the number of experimental trials should be small because the participant cannot repeatedly perform the sprint starts with maximal effort (no more than six trials) [27]. We could prepared only two conditions at the set position during the block start phase, and participant performed a total of six block-start trials. This was because of the unavoidable number of conditions as the block start experiment. Second, this study was conducted without the familiarization with the widened stance width in the block start. All participants were sprint specialists with enough training experience, suggesting that they were familiar with the set position in the normal condition relative to the widened condition. Even with extensive training in the widened condition before the experimental trial, the normal condition would be optimal to enhance the block-induced power relative to the widened condition. Despite these limitations, we have reported some new information that can serve as baseline data for future studies on developing the newly designed starting block.

In conclusion, a widened stance width at set position which we prepared in this study affected the hip-joint kinematics in both legs and hip power generation in the rear leg during the block start phase. However, when considering sprinting performance during the whole block start phase, there were no significant effect of the widened stance width on block-induced power and the subsequent sprint time.

## Supporting Information

S1 Video Trial in the widened condition. (WMV)

S2 Video Trial in the normal condition. (WMV)
